# Clinical Value of Surveillance ^18^F-fluorodeoxyglucose PET/CT for Detecting Unsuspected Recurrence or Second Primary Cancer in Non-Small Cell Lung Cancer after Curative Therapy

**DOI:** 10.3390/cancers14030632

**Published:** 2022-01-27

**Authors:** Chae Hong Lim, Soo Bin Park, Hong Kwan Kim, Yong Soo Choi, Jhingook Kim, Yong Chan Ahn, Myung-ju Ahn, Joon Young Choi

**Affiliations:** 1 Department of Nuclear Medicine, Soonchunhyang University Hospital Seoul, Soonchunhyang University College of Medicine, Seoul 04401, Korea; 100819@schmc.ac.kr (C.H.L.); soobin.park@schmc.ac.kr (S.B.P.); 2 Department of Thoracic and Cardiovascular Surgery, Samsung Medical Center, Sungkyunkwan University School of Medicine, Seoul 06351, Korea; hkts.kim@samsung.com (H.K.K.); ysooyah.choi@samsung.com (Y.S.C.); jhingook.kim@samsung.com (J.K.); 3 Department of Radiation Oncology, Samsung Medical Center, Sungkyunkwan University School of Medicine, Seoul 06351, Korea; ycahn.ahn@samsung.com; 4 Division of Hematology-Oncology, Department of Medicine, Samsung Medical Center, Sungkyunkwan University School of Medicine, Seoul 06351, Korea; silk.ahn@samsung.com; 5 Department of Nuclear Medicine, Samsung Medical Center, Sungkyunkwan University School of Medicine, Seoul 06351, Korea

**Keywords:** non-small cell lung cancer, ^18^F-fluorodeoxyglucose, PET/CT, surveillance, recurrence, second primary cancer

## Abstract

**Simple Summary:**

Non-small cell lung cancer (NSCLC) patients are at considerable risk of recurrence or second primary cancer (SPC) after curative therapy. The utility of ^18^F-fluorodeoxyglucose (FDG) positron emission tomography/computed tomography (PET/CT) surveillance to detect recurrent lesions in NSCLC patients without suspicion of recurrence has not been established. The aim of our retrospective study was to evaluate the diagnostic value of surveillance FDG PET/CT for detecting clinically unsuspected recurrence or SPC in patients with NSCLC after curative therapy. In a cohort of 2684 NSCLC patients after curative therapy, surveillance FDG PET/CT showed good diagnostic efficacy for detecting clinically unexpected recurrence or SPC. Furthermore, the diagnostic performance was improved in subgroups of patients with advanced stage prior to curative therapy, PET/CT scans performed within 3 years after curative-intent therapy, and curative surgery. Surveillance PET/CT can be more useful when performed soon after therapy in curative surgery recipients and those with an advanced disease stage considering its diagnostic efficacy and yield.

**Abstract:**

We evaluated the diagnostic value of ^18^F-fluorodeoxyglucose (FDG) positron emission tomography (PET)/CT surveillance for detecting clinically unsuspected recurrence or second primary cancer (SPC) in patients with non-small cell lung cancer (NSCLC) after curative therapy. A total of 4478 surveillance FDG PET/CT scans from 2864 NSCLC patients without suspicion of recurrence after curative therapy were reviewed retrospectively. In 274 of 2864 (9.6%) patients, recurrent NSCLC or SPC was found by surveillance PET/CT during clinical follow-up. Surveillance PET/CT scans showed sensitivity of 98.9% (274/277), specificity of 98.1% (4122/4201), accuracy of 98.2% (4396/4478), positive predictive value (PPV) of 77.6% (274/353), and negative predictive value of 99.9% (4122/4125). The specificity and accuracy in the curative surgery group were significantly higher than those in the curative radiotherapy group. PPV was significantly improved in subgroups of patients with advanced stage prior to curative therapy, PET/CT scans performed within 3 years after curative-intent therapy, and curative surgery. FDG PET/CT surveillance showed good diagnostic efficacy for detecting clinically unexpected recurrence or SPC in NSCLC patients after curative therapy. It can be more useful when performed soon after therapy in curative surgery recipients and those with an advanced disease stage considering its diagnostic efficacy and yield.

## 1. Introduction

Lung cancer is one of the most common cancers and the first leading cause of cancer-related death worldwide [1]. Non-small cell lung cancer (NSCLC) represents the majority of lung cancer cases, comprising about 85% of newly diagnosed lung cancer [2]. Despite advances in treatment, NSCLC patients remain at considerable risk of recurrence [3,4]. Recent studies reported that NSCLC patients with limited recurrent lesions at the time of detection might have the potential for overall survival benefit with local ablative therapy [5,6]. Therefore, early detection using routine image-based surveillance might lead to better clinical outcomes in NSCLC patients. On the basis of this concept, the National Comprehensive Cancer Network (NCCN) guidelines currently recommend chest CT surveillance with or without a contrast agent every 6–12 months for 2 years. 

^18^F-Fluorodeoxyglucose (FDG) positron emission tomography/computed tomography (PET/CT) is a valuable imaging modality used for staging, therapy assessment, and prognosis in many oncology patients [7,8]. A considerable number of studies demonstrated the diagnostic usefulness of PET/CT in patients with suspected NSCLC recurrence [9,10]. However, the utility of FDG PET/CT surveillance in detecting recurrent lesions in NSCLC patients without any clinical symptoms or other abnormal imaging findings has not been established [11]. While some previous studies reported an excellent diagnostic performance of 96–97% for FDG PET/CT surveillance [12,13], others failed to demonstrate its improved diagnostic efficacy compared to conventional imaging [14,15]. The contrary findings of these previous studies might have resulted from modest sample sizes and heterogeneous populations with different stages and curative modalities. Therefore, FDG PET/CT is not recommended for surveillance after curative-intent therapy.

In Korea, FDG PET/CT surveillance in cancer patients after curative therapy without suspicion of recurrence was covered by National Healthcare Insurance between 2006 and 2016 [16]. The aim of this retrospective study was to investigate the diagnostic value of surveillance FDG PET/CT for detecting clinically unsuspected recurrence or newly developed second primary cancer in patients with NSCLC undergoing curative therapy.

## 2. Materials and Methods 

### 2.1. Subject Identification

We retrospectively reviewed the medical records of NSCLC patients who underwent FDG PET/CT surveillance between 2006 and 2016 at our institute after curative treatment. During this time period, physicians at our institution ordered FDG PET/CT to confirm residual NSCLC within 6 months of curative therapy (residual post-treatment PET/CT) to evaluate the extent of relapsed disease after confirmation of recurrent NSCLC (relapsed restaging PET/CT) or to detect recurrent NSCLC in those without clinical symptoms or other abnormal findings later than 6 months after curative treatment (routine surveillance PET/CT) on the basis of Korean health insurance coverage. Our study included 3035 NSCLC patients who had no evidence of disease recurrence within 6 months following curative treatment and underwent at least one surveillance FDG PET/CT from 6 months to 5 years after their first curative treatment.

Among these candidates, 171 were excluded for the following reasons: presence of previous cancer during the 5 years before NSCLC diagnosis or coexisting primary malignant lesions at the initial diagnosis of NSCLC (*n* = 121 patients), inadequate documentation at first diagnosis (*n* = 28 patients), and inadequate follow-up duration of fewer than six months without histologic confirmation for abnormal findings (*n* = 22 patients). Consequently, a total of 2864 patients and their 4478 FDG PET/CT scans performed only for routine surveillance were included in this study. This retrospective cohort study was approved by our institutional review board. The requirement for written consent was waived.

### 2.2. FDG PET/CT Protocol 

All patients fasted for at least 6 h and had a blood glucose level < 200 mg/dL at the time of PET/CT. Imaging was performed 60 min after injection of 5 MBq/kg FDG (without intravenous or oral contrast) on a Discovery LS (GE Healthcare, Waukesha, WI, USA) or a Discovery STe PET/CT scanner (GE Healthcare). Continuous spiral CT was performed using an 8-slice helical CT (140 keV; 40–120 mA; Discovery LS) or 16-slice helical CT (140 keV; 30–170 mA; Discovery STe). An emission scan was performed from head to thigh for 4 min per frame in 2-D mode. Reconstruction of these attenuation-corrected PET images (4.3 × 4.3 × 3.9 mm) was performed using an ordered-subset expectation maximization algorithm (28 subsets, 2 iterations; Discovery LS). Alternatively, the emission scan was performed for 2.5 min per frame in 3-D mode, with reconstruction of the attenuation-corrected PET images (3.9 × 3.9 × 3.3 mm) performed using a 3-D ordered-subset expectation maximization algorithm (20 subsets, 2 iterations; Discovery STe).

### 2.3. FDG PET/CT Imaging Report Review 

All PET/CT reports were reviewed retrospectively to detect possible recurrent or new second primary malignant lesions by one nuclear medicine physician blinded to patient outcome. At our institution, the interpretations of all PET/CT reports were by the consensus of two nuclear medicine physicians (one of them being an imaging specialist with more than 10 years of experience in nuclear medicine). Imaging interpretation was based on visual inspection with semiquantitative analyses and comparisons with prior FDG PET/CT studies. Sites of abnormal metabolic activity were classified as either suspected malignant or benign by applying imaging criteria. General rules of criteria for suspected malignant lesions were as follows: (1) Focal abnormally increased FDG uptake that exceeded that of the surrounding tissue and was discernible from physiological uptake or benign conditions was considered as a possible malignant condition. (2) In the brain, reduced glucose metabolism relative to that in the surrounding tissue was considered an abnormal finding. (3) Focal FDG activity in the primary malignant site after definitive radiotherapy or in the resection site after curative surgery was deemed as possible local recurrence. (4) Regarding the intrathoracic lymph nodes, FDG avid nodes symmetrically visualized in bilateral pulmonary hilar or mediastinal area with calcification or high CT attenuation were interpreted as benign [17]. However, new or interval increased FDG avid nodes compared with the prior PET/CT scan were interpreted as suspected metastatic lymphadenopathy. (5) Discrete pulmonary nodules or nodular opacity with significant FDG uptake was classed as recurrent lesion or new primary lung cancer. Non-clustered pulmonary nodules were classified as possible recurrent lesions regardless of the presence of FDG uptake. (6) Incidental focal FDG uptake in the breast, upper respiratory tract, gastrointestinal tract, thyroid gland, prostate gland, and salivary gland were separately classified as possible second primary malignancies. 

### 2.4. Medical Record Review and Clinical Decision

Clinical information, tumor characteristics, and treatment history at initial presentation, in addition to relapsed information during clinical follow-up, were obtained from medical records. Information about the first recurrent lesions within 5 years of curative therapy was investigated in all included subjects. Recurrence of NSCLC was diagnosed by pathological confirmation or a clinical decision based on imaging. When recurrence was diagnosed within six months of a positive PET/CT scan, it was designated a true-positive scan. When recurrence was not diagnosed for six months after a negative PET/CT scan, it was designated a true negative. If a possible recurrent lesion mentioned on PET/CT had a biopsy-confirmed benign histological finding or if follow-up imaging studies were consistent for at least 6 months, it was judged to be a false-positive result. If recurrence was confirmed within six months after negative PET/CT scans, the scans were regarded as false negatives. Second primary malignancy was determined by pathological confirmation. Persistently observed ground glass or sub-solid nodules without interval change were not considered in this analysis because they were not detected by surveillance PET/CT, and the relevant therapeutic decision is made over the long term.

At the first presentation of a relapsed site, the pattern of recurrence and the extent of disease were evaluated. The recurrent patterns were classified as loco-regional or distant recurrence on the basis of FDG PET/CT. Distant metastasis with coexisting loco-regional recurrence was categorized separately as combined recurrence. Multiple distant recurrences included recurrent lesions in multiple organs or multiple lesions in a single organ. The remaining distant recurrence was considered as oligo-distant metastasis in a solitary site. A possibly new primary lung cancer was included as part of the loco-regional recurrent pattern. 

### 2.5. Follow-Up Scheme

NSCLC patients were scheduled for routine follow-up every 3–6 months for the first 3 years after curative-intent therapy, then every 6 months from year 4–5 [17]. Chest CT scan was routinely used as the surveillance imaging modality. The curative surgery recipients with advanced disease stage or adjuvant treatment underwent additional PET/CT scans to confirm no evidence of disease within 6 months after the operation. These post-treatment PET/CT scans were also obtained from 3–6 months following completion of definitive radiotherapy or chemoradiotherapy. In those without evidence of disease, routine surveillance FDG PET/CT was performed replacing CT every 1–2 years from 6 months following curative-intent therapy. They were performed without any cancer-related therapy during the follow-up and used more frequently in the high-risk population with advanced stage. In case of a positive result for surveillance FDG PET/CT, tissue confirmation or a close follow-up including FDG PET/CT was performed to identify the presence of relapsed disease.

### 2.6. Statistical Analysis

The diagnostic performance of PET/CT scans for recurrent NSCLC was evaluated by calculating the sensitivity, specificity, positive predictive value (PPV), negative predictive value (NPV), and accuracy. Subgroup analyses based on initial stage, follow-up duration, and type of curative-intent treatment were performed. A chi-square test or Fisher’s exact test was used for comparisons. All statistical analyses were performed with SPSS^®^ version 24.0 for Windows (Chicago, IL, USA), and *p* values < 0.05 were considered significant.

## 3. Results 

### 3.1. Characteristics of the Study Subjects

Demographic, tumor, and treatment characteristics of the 2864 patients are listed in Table 1. There were 1914 men and 950 women, ranging in age from 19 to 89 years (mean ± SD, 61.1 ± 9.8 years). The histological type was adenocarcinoma in 1836 cases (64.1%), squamous cell carcinoma in 855 cases (29.9%), and other NSCLC in 173 cases (6.0%). Based on the seventh edition of the TNM cancer staging system, 46.5% of cases were stage I, 30.7% were stage II, 20.2% were stage IIIA, and 2.6% were stage IIIB. The primary curative treatment was surgery in 2711 patients (94.7%), radical radiotherapy with concurrent chemotherapy in 77 (2.7%), and definitive radiotherapy in 76 (2.6%). Neoadjuvant treatment was given to 249 patients (8.7%), and adjuvant treatment was performed with 1062 patients (37.1%). There was one surveillance PET/CT scan in 1746 patients (61.0%) and two surveillance scans in 730 patients (25.5%).

### 3.2. Diagnostic Performance of Surveillance FDG PET/CT for Detection of Relapse 

During all clinical follow-ups, 277 cases of recurrent NSCLC were confirmed within 6 months after a surveillance PET/CT. Among 4478 surveillance PET/CT scans, 353 (7.9%) had positive results for recurrence. Of 353 positive scans, 274 proved to be true-positive (129 by histology and 145 by imaging or follow-up), whereas the remaining 79 scans were found to be falsely positive. Among 4125 PET/CT scans with negative results, 4122 had true-negative results. In the remaining three cases, classified as false negatives, recurrent diseases were detected within six months after negative PET/CT scans (one by histology, two by imaging or follow-up). Therefore, the diagnostic efficacy of surveillance PET/CT scans for detecting recurrent NSCLC was represented by a sensitivity of 98.9% (274/277), specificity of 98.1% (4122/4201), accuracy of 98.2% (4396/4478), PPV of 77.6% (274/353), and NPV of 99.9% (4122/4125) (Table 2).

### 3.3. Comparison of Diagnostic Capability of Surveillance FDG PET/CT According to Clinical Setting

The diagnostic performances of surveillance FDG PET/CT were compared between subgroups classified according to clinical settings, including initial stage of disease, PET/CT timing after curative-intent therapy, and initial curative treatment modality (Table 2).

The incidence of recurrent NSCLC detected by surveillance PET/CT was significantly increased according to stage of disease (stage I, 3.4%; stage II, 5.8%; stage III, 12.7%; *p* < 0.001) and was significantly decreased according to the time interval between curative therapy and surveillance PET/CT (<12 mo., 7.7%; 12–36 mo., 6.5%; ≥36 mo., 2.8%; *p* < 0.001). However, there was no significant difference in the incidences between curative modalities (surgery, 6.1%; RT ± CTx, 7.7%; *p* = 0.330).

Regarding the subgroup analysis for diagnostic performance, there were no significant differences in the sensitivity (97.6–100.0%) and NPV (99.9–100.0%) of surveillance PET/CT according to initial stage, time interval between curative therapy and PET/CT, or treatment modality. Although the specificity (97.7–98.5%) and accuracy (97.9–98.6) were not significantly different according to initial stage and time interval between curative therapy and PET/CT, they were significantly higher in the subgroup with curative surgery than in the subgroup with curative radiotherapy (specificity, 98.4 vs. 93.1, *p* < 0.001; accuracy, 98.4 vs. 93.6, *p* < 0.001). The PPV was significantly increased according to stage (stage I, 68.6%; Stage II, 74.8%; Stage III, 86.1%; *p* = 0.004) and was significantly decreased when surveillance PET/CT was performed at ≥36 mo. compared to at <12 mo. or 12–36 mo. after therapy (<12 mo., 79.0%; 12–36 mo., 82.3%; ≥36 mo., 56.8%; *p* = 0.002). The PPV was significantly higher in the subgroup that received curative surgery than the radiotherapy subgroup (surgery, 79.8%; RT ± CTx, 54.8%; *p* = 0.002).

### 3.4. Diagnostic Performance of Surveillance FDG PET/CT according to Initial Stage and Timing of PET/CT Scan after Curative-Intent Therapy 

In order to eliminate possible bias due to heterogeneity in the incidence rate of recurrent event, subjects were divided into four subgroups according to initial stages (stage I–II vs. stage III) and interval times of PET/CT (<36 mo. vs. ≥36 mo.). Diagnostic performances according to respective subgroups are shown in Table 3. In all subgroups, surveillance PET/CT showed high sensitivity, specificity, NPV and accuracy, which were not significantly different. The PPV was significantly higher in the subgroup with advanced stage at early timing after curative-intent therapy compared to those with early stage at early (86.5% vs. 76.1%, *p* = 0.023) or late timing (86.5% vs. 48.5%, *p* < 0.001). The PPV in the subgroup with early stage and early timing was significantly higher than that in the subgroup with early stage and late timing (76.1% vs. 48.5%, *p* = 0.001). Although the PPV of the subgroup with advanced stage and late timing was higher than that of the subgroup with early stage and late timing, it showed borderline statistical significance (81.8% vs. 48.5%; *p* = 0.056).

### 3.5. The Extent of First Recurrence Detected by True-Positive Surveillance PET/CT Results 

**Figure 1:**
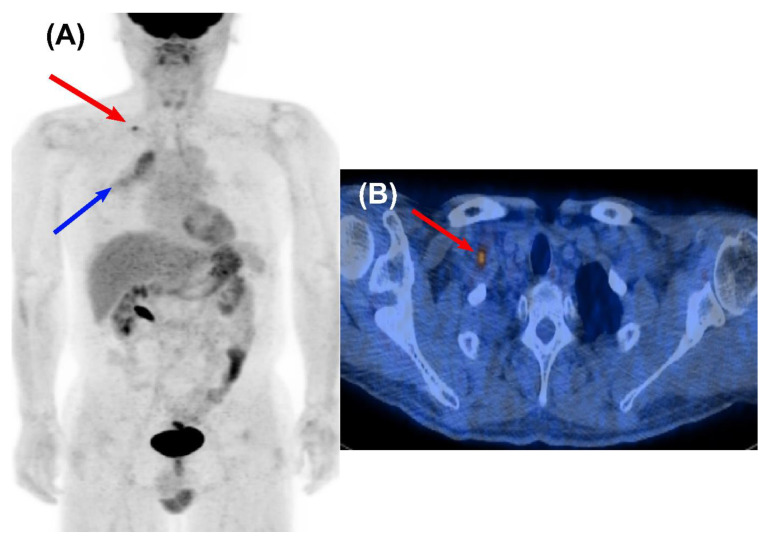
Loco-regional recurrence detected by surveillance FDG PET/CT in a 54-year-old male who underwent curative surgery for NSCLC. Focal FDG uptake was noted in the right supraclavicular lymph node on a maximum intensity projection (MIP) (**A**) and a transaxial fused PET/CT image (**B**) obtained at 7 months after curative resection. The lesion was confirmed as biopsy-proven recurrence (red arrow). He survived for 60 months after localized treatment for the lesion. Diffusely increased FDG uptake on the right upper lung field indicates radiation pneumonitis (blue arrow).

**Figure 2:**
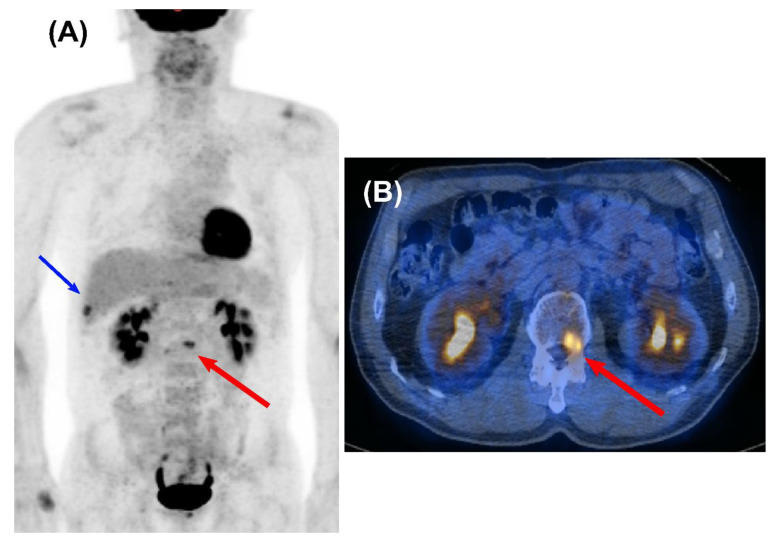
Oligometastasis detected by surveillance FDG PET/CT in a 58-year-old male who underwent curative surgery for NSCLC. A maximum intensity projection (MIP) (**A**) and a transaxial fused PET/CT image (**B**) obtained at 16 months after curative resection showed focal FDG uptake on the L2 spine, indicating metastasis to the bone (red arrow). He survived for 70 months after localized radiotherapy with palliative chemotherapy. Another focal FDG uptake on the right lower thorax indicates recent post-traumatic change (blue arrow).

Of the 274 true relapses that were detected by surveillance PET/CT, the disease extent of recurrence was 144 (52.6%) in loco-regional recurrence (Figure 1), 88 (32.1%) in distant recurrence (Figure 2), and 42 (15.3%) in both. Among the 88 patients with distant-only recurrences, oligo-distant metastasis to a solitary site (52.3%) was more frequent than other multiple distant metastasis (47.7%).

### 3.6. Imaging Characteristics of False-Positive and False-Negative Surveillance PET/CT Results

In a total of 79 false-positive cases, there were 38 (48.0%) in the lung, 30 (38.0%) in the lymph nodes, and 11 (14.0%) in other organs. In the lung, pulmonary nodular lesions (31.6%) were most frequently misinterpreted as possibly malignant, followed by avid uptake of FDG at postoperative (10.1%) and post-radiotherapy sites (6.3%). False-positive hypermetabolic lymph nodes were distributed mainly in thoracic regions, including the pulmonary hilar and mediastinal zone (20.3%) and the supraclavicular zone (15.2%). Two cases (2.5%) involved either the axillary space or abdominal mesentery, respectively. Among distant organs, which included the spine (*n* = 2), rib (*n* = 1), and femoral head (*n* = 1), solitary bone lesions with focal FDG uptake were interpreted commonly as suspected metastases. Other organ sites with false-positive FDG uptake were the liver (*n* = 3), muscle (*n* = 1), and adrenal gland (*n* = 1). Two cases with hypometabolic brain lesions showed false-positive results. Three cases showed recurrent NSCLC within six months after negative PET/CT scans. One patient developed pleural metastasis, which was identified by the presence of tumor cells in pleural fluid. Another patient had multiple metastatic pulmonary nodules. The other patient showed solitary rib metastasis and pleural metastasis in follow-up imaging studies.

### 3.7. Detection of Clinically Unsuspected Second Primary Cancer 

**Figure 3:**
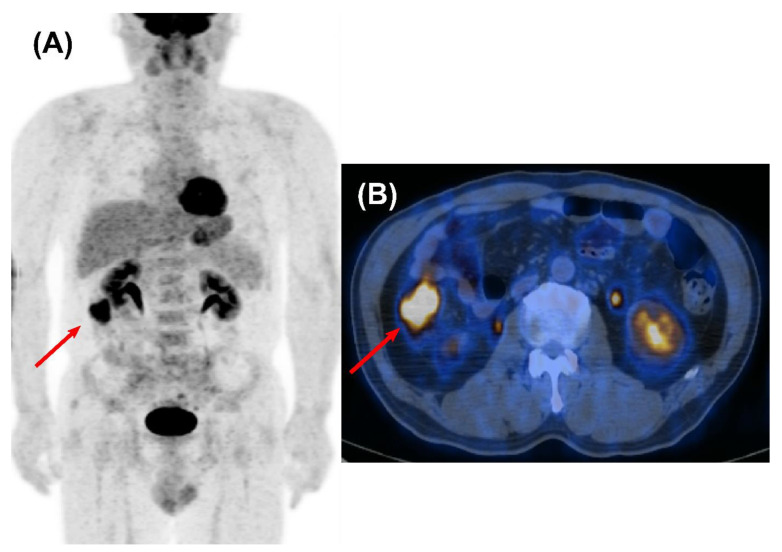
Second primary colon cancer detected by surveillance FDG PET/CT in a 52-year-old male who underwent curative surgery for NSCLC. A maximum intensity projection (MIP) (**A**) and a transaxial fused PET/CT image (**B**) obtained at 4 years after curative resection demonstrated a hypermetabolic lesion on the ascending colon without evidence of metastasis (red arrow). It was identified as primary colon cancer after surgical resection, and he has survived for more than 10 years after initial diagnosis of NSCLC.

In 4478 surveillance PET/CT scans, 82 (1.8%) showed possible second primary malignancy in extra-pulmonary regions (Figure 3). Among them, 35 cases were diagnosed as second primary malignancies by tissue confirmation. The most frequent location was colon and rectum (*n* = 9), followed by the thyroid (*n* = 8), head and neck (*n* = 4), prostate (*n* = 4), hepatobiliary region (*n* = 2), pancreas (*n* = 2), uterine cervix (*n* = 1), stomach (*n* = 1), and breast (*n* = 1). The remaining three cases were hematologic malignancies, including lymphoma. Those clinical stages corresponded to early stages confined to primary tumor in 30 cases (85.7%), locally advanced initial stages with primary tumor and regional lymph nodes in four cases (11.4%), and advanced initial stage with distant metastasis in only one case. There were no cases of second primary malignancy detection within 6 months after negative PET/CT. The PPV of surveillance PET/CT scans was 42.7% (35/82). When considering the respective organ sites, the PPV was 100% for hematological (3/3), hepatobiliary (2/2), uterine cervix (1/1), and breast (1/1) malignancies, followed by 69.2% in colorectal malignancies (9/13), 44.4% in head and neck malignancies (4/9), 40.0% in pancreas malignancies (2/5), 33.0% in upper gastrointestinal malignancies (1/3), 27.6% in thyroid malignancies (8/29), and 25.0% in prostate malignancies (4/16).

## 4. Discussion

The present study focused on the diagnostic performance of surveillance FDG PET/CT for detecting clinically unsuspected recurrence or SPC in NSCLC patients after curative therapy. Excellent diagnostic efficacy was found, including a sensitivity of 98.9%, specificity of 98.1%, and accuracy of 98.2%. Recurrent lesions, especially, detected by surveillance FDG PET/CT were distributed more commonly in loco-regional sites, where local salvage therapy can provide a second chance for remission. Considering the incidence of positive PET/CT and PPV, surveillance FDG PET/CT might be more useful in subgroups with advanced stage prior to curative therapy, PET/CT scans performed <3 years after curative-intent therapy, and curative surgery.

A large number of studies demonstrated the clinical usefulness of FDG PET/CT through its excellent diagnostic performance for initial staging or detection of suspicious recurrence in NSCLC patients [9,12,18]. However, the diagnostic advantage of surveillance PET/CT in NSCLC patients without suspicion of recurrence remains controversial. The main problem is that previous studies failed to show a consistent result regarding the diagnostic value of surveillance FDG PET/CT, despite its expensive cost [13,14,15,19]. This might be because previous studies were performed with an insufficient number of NSCLC patients with different stages or curative types. We enrolled 2864 NSCLC patients owing to the broad coverage of our national healthcare insurance system [16]. Our large population-based study showed favorable results, demonstrating a high diagnostic accuracy of about 98% for surveillance PET/CT. Further subgroup analyses according to stage and curative type were performed to exclude the potential influence of population heterogeneity due to the retrospective design. The results showed that a high accuracy was not significantly different according to initial stage. However, surveillance PET/CT in NSCLC patients that received curative radiotherapy showed a relatively lower diagnostic accuracy of about 93%, which might be related to the false-positive results possibly associated with active inflammation due to radiation therapy. Our results correspond to previous results in that diagnostic performance was better in postoperative NSCLC patients that received curative surgery compared to radiotherapy [13,14,15,19].

The second issue regarding the diagnostic value of surveillance PET/CT is its cost-effectiveness, whether it can result in the improvement of survival despite its high cost [8]. Previous studies reported that frequent routine surveillance of NSCLC patients after curative-intent therapy did not improve overall and post-recurrence survival [20]. Such results led CT to be chosen as a surveillance imaging modality due to its relatively low cost compared to PET/CT. However, recent studies reported that the aggressive treatment of early recurrence has the potential for overall survival benefit in NSCLC patients in conjunction with improving local and systemic salvage options [5,6]. In this trend, FDG PET/CT can be a better imaging modality than conventional anatomical imaging for early detection of recurrent lesions in NSCLC patients because cancer-related metabolic abnormalities usually precede structural changes [8,10,21]. The present study indicated high sensitivity of FDG PET/CT in the surveillance follow-up of NSCLC patients, where only three out of 277 patients that relapsed within six months after surveillance PET/CT were missed. A prior meta-analysis has highlighted the improved accuracy of PET/CT compared to standard CT, mainly due to its improved sensitivity [18]. In addition, recurrent lesions detected by our surveillance FDG PET/CT were more commonly distributed in loco-regional sites, unlike previous studies which reported that relapsed NSCLC showed distant metastasis more frequently at the time of detection [22,23]. These findings indicate that surveillance PET/CT may offer an appropriate chance for local salvage therapy. Further studies are needed to investigate whether the early detection of surveillance FDG PET/CT has survival benefit in relapsed NSCLC patients.

The third concern regarding the clinical utility of surveillance PET/CT is a low positive predictive value due to false-positive FDG avid lesions, which can cause high economic burdens by recommending unnecessary further evaluations. Gambazzi et al. reported a relatively low PPV of 56% for detecting NSCLC recurrence in surveillance PET/CT [19]. The PPV of our study was higher than that of previous research, but it remained suboptimal (77.6%). However, PPV was significantly improved to 86.5% in subgroups who had initial advanced stage and underwent surveillance PET/CT within 3 years after curative-intent therapy. The PPV of the subgroup with advanced stage and late timing was also relatively high, compared to that of overall subject (81.8% vs. 77.6%). In contrast, PPV was poor (48.5%) in subgroups with early-stage disease at a late time after curative-intent therapy. This result demonstrates that risk factors for recurrence can have a considerable effect on PPV in the surveillance setting with relatively lower disease prevalence [24]. A recent study by Toba et al. reported that it might be sufficient to perform follow-up PET/CT until 3 years after the operation, especially for advanced stage patients [25]. However, it included only six surveillance PET/CT scans performed after 4 years post-resection in NSCLC patients with initial advanced stage. Furthermore, recurrent events occurred more frequently in the stage I–II patients than in those with advanced stage after 4 years post resection. A relatively small sample size may be inadequate for the evaluation of the effectiveness of surveillance PET/CT scans according to timing after curative-intent therapy. Consequently, our results suggest that surveillance PET/CT may not be cost-effective in low-risk populations at late times after curative-intent treatment. In the future, the approach considering risk stratification may be important for an efficient strategy of surveillance protocol, including FDG PET/CT, in NSCLC patients.

In FDG PET/CT scans, another main cause of false-positive results was post-therapeutic effects, such as those following surgical resection or radiotherapy. These post-therapeutic inflammatory changes generally decrease at 6 months after curative-intent treatment [13]. However, a few studies mentioned the long-term effects of post-therapeutic changes, where pulmonary lesions were sometimes described as nodular lesions, such as suture granuloma, granulomatous inflammatory lesion, or organizing pneumonia [26,27]. The post-therapeutic effect also can be associated with reactive lymphadenitis after curative-intent treatment [28]. Our false-positive lesions were distributed more commonly in thoracic regions that received surgery and radiotherapy compared to extra-thoracic sites (83.6% vs. 16.4%). Most of those lesions were surgical stumps, pulmonary nodules, or intrathoracic lymph nodes. These distributed characteristics of false-positive cases showed similar results to prior research [15]. The higher radiation dose to the lung in curative radiotherapy, especially, could also have a greater impact on such findings [29]. In this regard, those with prior curative radiotherapy history showed significantly lower PPV (54.8% vs. 79.8%) compared to those without such medical history. 

Several indicators can be helpful to distinguish true-positive lesions from FDG-avid sites. Among those, SUV has been commonly used as an objective parameter to characterize suspicious high uptakes. However, some benign diseases showed considerable overlaps in FDG uptake levels. In these cases, CT features as signs of benignity regardless of FDG avidity reduced the number of false-positive cases compared to using SUVmax alone, as described in our previous study [30]. On the other hand, anatomical changes in CT images sometimes may be more important than FDG accumulation for detecting relapse disease. Actually, SUV could be underestimated in malignant pleural effusion with small cell component, small pulmonary nodule, or ground-glass lesion [31,32]. Three false-negative cases of this study were also pleural and pulmonary metastasis, which could be found in follow-up chest CT. Additional contrast-enhanced CT (ceCT) may also serve as a complementary tool by discerning vessel or bowel activity [33]. However, the increased cost and radiation exposure can outweigh the perceived benefit. There are also no studies demonstrating the definite advantage for performing both PET/CT and ceCT for the purpose of cancer surveillance. For these reasons, the addition of ceCT to the PET/CT protocol has been not covered by health insurance in some countries, including our country. Furthermore, some previous studies reported that the PPV of surveillance FDG PET/CT was higher than that of surveillance chest CT [34,35]. As such, the NCCN guidelines currently recommend the use of FDG PET/CT to differentiate true malignancy from an inconclusive result on standard CT during curative NSCLC patient follow-up. 

A previous study reported that new second primary cancer in NSCLC patients could be associated with unfavorable prognosis [36]. Thus, early detection and management of new second primary cancer might improve survival in NSCLC patients. FDG PET/CT can be helpful in detecting second primary malignancies in NSCLC patients [37,38]. Although the most common site of second primary cancer in NSCLC patients is the lung, the definition of second primary lung cancer remains controversial. This can lead to inaccuracies in rates of recurrence and second primary lung cancer [39]. Hence, our study did not separate recurrent pulmonary lesions and second primary lung cancer. Excluding second primary lung cancer, surveillance PET/CT detected 35 unexpected second primary malignancies in extra-pulmonary regions. The most common type of second primary extra-pulmonary malignancy was colorectal cancer, which was consistent with a previous study performed in a large population of NSCLC patients [36]. Other second primary malignancies were identified in the thyroid, head and neck, prostate, hepatobiliary, pancreas, uterine cervix, breast, and stomach. Although the PPV was low (42.7%), 33 of 52 false-positive cases were located in the prostate gland or thyroid gland, which are well-known false-positive sites [40,41]. Most true-positive cases were located outside the field of view of chest CT images and corresponded to early stages at the time of detection, with the expectation of remission after therapy. Appropriate addition of FDG PET/CT in the surveillance setting for NSCLC patients can be helpful for early detection of second primary malignancy as well as recurrence, which may improve the survival rate of NSCLC patients after curative-intent treatment.

This retrospective study has several limitations. First, the follow-up period and frequency of surveillance PET/CT after curative-intent treatment were decided according to the preference of the clinicians because there is no consensus regarding the utility of surveillance PET/CT. As a result, the interval period and frequency of surveillance FDG PET/CT varied between subjects. However, further subgroup analyses according to the follow-up period and tumor stage complements the problem of heterogeneity in our populations. Second, the inherent limitation of the retrospective design with selection bias might lead to diagnostic test bias [42]. Therefore, a prospective study will be required to validate the conclusions of this study. Third, surveillance PET/CT was not performed concurrent with chest CT due to the radiation exposure and cost issues. Hence, we could not directly compare the diagnostic performance of surveillance PET/CT and chest CT. However, PET/CT generally is reported to have better diagnostic performance than chest CT for diagnosis of recurrent NSCLC. Furthermore, about half of recurrent cases detected by PET had distant metastasis, which would not have been covered by chest CT. Fourth, this study was analyzed using imaging reports interpreted by several nuclear medicine physicians, which could cause inter-observer variability. It might improve inter-observer variability if one reader were to directly review all PET/CT images. However, some abnormalities can be missed and misinterpreted by this method, especially in a study with a large amount of image data, such as this one [43]. These errors could be decreased with double reading per FDG PET/CT scan used in our imaging reports system [44]. Finally, our study did not perform additional analysis for survival benefit. Our results were not conclusive regarding the cost-effectiveness of surveillance PET/CT. A previous prospective study reported that surveillance PET/CT were potentially cost-effective after curative-intent treatment in NSCLC patients [45]. However, the authors acknowledged the limitation of not considering the chance of overestimating the cost-effectiveness caused by false-positive findings. Further study is warranted.

## 5. Conclusions

Surveillance FDG PET/CT showed good diagnostic efficacy for detecting clinically unexpected recurrence and SPC in NSCLC patients after curative therapy. Although the incidence of positive PET/CT was not high, considering that its high diagnostic performance and detection of early loco-regional recurrence and SPC could provide opportunities for early therapeutic intervention and subsequent cure, prompt and routine use of surveillance PET/CT should be considered, especially for those with advanced stage disease or undergoing curative surgery. Further randomized prospective studies are needed to evaluate the survival benefit and cost-effectiveness of surveillance PET/CT compared to other imaging modalities.

## Figures and Tables

**Table 1:** Characteristics of 2684 NSCLC patients who underwent 4478 surveillance FDG PET/CT scans.

Variable		Number (%)
Age, years	Mean ± SD	61.1 ± 9.8
Sex	Male	1914 (66.8%)
Location	Right lung	1670 (58.3%)
	Left lung	1194 (41.6%)
Histology	Adenocarcinoma	1836 (64.1%)
	Squamous cell carcinoma	855 (29.9%)
	Other NSCLC	173 (6.0%)
T stage	T1	1324 (46.2%)
	T2	1203 (42.0%)
	T3	243 (8.5%)
	T4	94 (3.3%)
N stage	N0	1962 (68.5%)
	N1	365 (12.7%)
	N2	484 (16.9%)
	N3	53 (1.9%)
Stage	I	1333 (46.5%)
	II	879 (30.7%)
	IIIA	577 (20.2%)
	IIIB	75 (2.6%)
Curative treatment modality	Surgery	2711 (94.7%)
	Definitive CCRT	77 (2.7%)
	Definitive RT	76 (2.6%)
Neoadjuvant treatment	No	2618 (91.4%)
	CCRT	199 (7.0%)
	RT	6 (0.2%)
	Chemotherapy	44 (1.5%)
Adjuvant treatment	No	1802 (62.9%)
	CCRT	242 (8.5%)
	RT	169 (5.9%)
	Chemotherapy	648 (22.6%)
Number of surveillance PET/CT	1	1746 (61.0%)
scans for each patient	2	730 (25.5%)
	3	294 (10.3%)
	4	80 (2.8%)
	5	14 (0.5%)

The stages were based on the American Joint Committee on Cancer (AJCC) Staging Manual, seventh edition; FDG, ^18^F-fluorodeoxyglucose; PET/CT, positron emission tomography/computed tomography; NSCLC, non-small cell lung cancer; SD, standard deviation; CCRT, concurrent chemoradiotherapy; RT, radiotherapy.

**Table 2:** Diagnostic performance of surveillance FDG PET/CT scans for detection of recurrent NSCLC.

Parameter		Incidence of Recurrence	TP (N)	FP (N)	FN (N)	TN (N)	Sensitivity (%)	Specificity (%)	PPV (%)	NPV (%)	Accuracy (%)
Overall		6.2% (277/4478)	274	79	3	4122	98.9	98.1	77.6	99.9	98.2
Initial stage (AJCC 7th)	I	3.4% (70/2067)	70	32	0	1965	100.0	98.4	68.6	100.0	98.5
	II	5.8% (82/1427)	80	27	2	1318	97.6	98.0	74.8	99.9	98.0
	III	12.7% (125/984)	124	20	1	839	99.2	97.7	86.1	99.9	97.9
Time interval between curative therapy and PET/CT, months	<12	7.7% (130/1685)	128	34	2	1521	98.5	97.8	79.0	99.9	97.9
	12–36	6.5% (122/1893)	121	26	1	1745	99.2	98.5	82.3	99.9	98.6
	≥36	2.8% (25/900)	25	19	0	856	100.0	97.8	56.8	100.0	97.9
Curative treatment modality	Surgery	6.1% (260/4258)	257	65	3	3933	98.8	98.4	79.8	99.9	98.4
	RT ± CTx	7.7% (17/220)	17	14	0	189	100.0	93.1	54.8	100.0	93.6

FDG, ^18^F-fluorodeoxyglucose; PET/CT, positron emission tomography/computed tomography; NSCLC, non-small cell lung cancer; TP, true positive; FP, false positive; FN, false negative; TN, true negative; PPV, positive predictive value; NPV, negative predictive value; RT, radiotherapy; CTx, chemotherapy; AJCC, American Joint Committee on Cancer.

**Table 3:** Diagnostic performance of surveillance FDG PET/CT scans in subgroups stratified according to initial stage and PET/CT timing after curative-intent therapy.

Parameter	Total (N)	TP (N)	FP (N)	FN (N)	TN (N)	Sensitivity (%)	Specificity (%)	PPV (%)	NPV (%)	Accuracy (%)
PET/CT scan < 36 with initial stage I–II	2757	134	42	2	2579	98.5	98.4	76.1	99.9	98.4
PET/CT scan < 36 with initial stage III	821	115	18	1	687	99.1	97.5	86.5	99.9	97.7
PET/CT scan ≥ 36 with initial stage I–II	737	16	17	0	704	100.0	97.6	48.5	100.0	97.7
PET/CT scan ≥ 36 with initial stage III	163	9	2	0	152	100.0	98.7	81.8	100.0	98.8

FDG, ^18^F-fluorodeoxyglucose; PET/CT, positron emission tomography/computed tomography; NSCLC, non-small cell lung cancer; TP, true positive; FP, false positive; FN, false negative; TN, true negative; PPV, positive predictive value; NPV, negative predictive value; RT, radiotherapy; CTx, chemotherapy; AJCC, American Joint Committee on Cancer.

## Data Availability

Restrictions apply to the availability of these data. Data were obtained from the Samsung Medical Center and are available from the corresponding author with the permission of the Samsung Medical Center.
